# 

**Published:** 1874-10

**Authors:** 


SOME PRACTICAL HINTS FOR THE TREATMENT AND THE PREVENTION OF DISEASES OF WOMEN. By William Goodell, M.D., Physician-in-charge of the Preston Retreat; Clinical Professor of the Diseases of Women and Children in the University of Pennsylvania, etc. Philadelphia: 1874. Octavo, paper, pp. 33.

---

ON THE MEANS EMPLOYED AT THE PRESTON RETREAT for the Prevention and Treatment of Puerperal Diseases. By Wm. Goodell, M.D., Physician-in-charge, etc. Philadelphia: 1874, Octavo, paper, pp. 15.

---

PRESIDENT'S ADDRESS before the Michigan State Medical Society. By Edward W. Jenks, M.D., Detroit, Mich. 1874. Octavo, paper, pp. 15.

CELL LIFE, the source of all power, both physical and mental, in the human economy. The annual oration, delivered before the Medical Assosiation of the State of Alabama. By S. D. Seelye, M.D., Montgomery, Alabama. 1874. Octavo, paper, pp. 31.

---

HÆMORRHAGIC MALARIAL FEVER, an address delivered before the Medical Society of North Carolina, by William A. B. Norcom, M.D., of Edenton, President of the Society. 1874. Octavo, paper, pp. 30.

---

EPIDEMIC OF YELLOW FEVER IN MONTGOMERY, ALABAMA, DURING THE SUMMER OF 1873, by R. F. Michel, M.D., of Montgomery. 1874. Octavo, paper, pp. 25.

---

REPORT OF CASES TREATED BY ELECTRICITY, and addenda of Electro-Medicine and Electro-Surgery, by John J. Caldwell, M.D., of Baltimore, Md. 1873. Pamphlet, pp. 24.

---

THE CONNECTION BETWEEN EXCESSIVE NERVE AND BRAIN WORRY AND BODILY DISEASE, with pathology and treatment of cancers, tumors, etc. By J. J. Caldwell, M.D. Baltimore: 1874. Pamphlet, pp. 7.

---

A REVIEW OF THE RECENT RESEARCHES IN THE PATHOLOGY AND TTEATMENT OF CANCER. By J. J. Caldwell, M.D. Baltimore: 1874. Pamphlet, pp. 8.

---

HYPODERMIC INJECTION OF ETGOT IN POST-PARTUM HEMORRHAGE. By P. C. Williams, M.D. Baltimore: 1874. pp. 4.

---

COCAIN, VERATRIA AND GELSEMIUM. TOXICOLOGICAL STUDIES. By I. Ott, Easton, Penn. Philadelphia: Lindsay & Blakiston. 1874. 12 mo., paper, pp. 53.

---

THE METEOR. Edited by a patient in the Alabama Insane Hospital; quarterly, October, 1874.

---

FIRST ANNUAL ANNOUNCEMENT OF THE COLLEGE OF PHYSICIANS AND SURGEONS OF INDIANA. Indianapolis: 1874.

Tennessee Pharmacal Gazette, a monthly review of Practical Pharmacy. Edited by Benjamin Lillard, Phar. D., September, 1874.

### ACKNOWLEDGMENTS.

Atlanta Daily Herald; Rome Daily Commercial; The Commonwealth; American Journal of Pharmacy; American Practitioner; Archives of American Medicine and Surgery; Boston Medical and Surgical Journal; Baltimore Physician and Surgeon; Buffalo Medical and Surgical Journal; The Clinic; The Druggist; The Galaxy; Herald of Health; Journal of Materia Medica; London Lancet; Louisville Medical Reporter; Medical Record; Medical News and Library; Medical and Surgical Reporter; Medical Times; Nashville Journal of Medicine and Surgery; Pacific Medical and Surgical Journal; Popular Science Monthly; Physician and Pharmacist; Supplement to Medical News and Library; Texas Medical Journal; Virginia Medical Monthly.

